# Insights from unifying modern approximations to infections on networks

**DOI:** 10.1098/rsif.2010.0179

**Published:** 2010-06-10

**Authors:** Thomas House, Matt J. Keeling

**Affiliations:** Department of Biological Sciences, Mathematics Institute, University of Warwick, Gibbet Hill Road, Coventry CV4 7AL, UK

**Keywords:** epidemic, network, transmission, pairwise, simulation, infection

## Abstract

Networks are increasingly central to modern science owing to their ability to conceptualize multiple interacting components of a complex system. As a specific example of this, understanding the implications of contact network structure for the transmission of infectious diseases remains a key issue in epidemiology. Three broad approaches to this problem exist: explicit simulation; derivation of exact results for special networks; and dynamical approximations. This paper focuses on the last of these approaches, and makes two main contributions. Firstly, formal mathematical links are demonstrated between several prima facie unrelated dynamical approximations. And secondly, these links are used to derive two novel dynamical models for network epidemiology, which are compared against explicit stochastic simulation. The success of these new models provides improved understanding about the interaction of network structure and transmission dynamics.

## 1. Introduction

The vast majority of infectious diseases can be considered as spread through a network of contacts between individuals or groups. These network concepts have been used, to great effect, in scenarios where the network of relevant contacts can be readily ascertained: for sexually transmitted infections, the network of human sexual contacts [1–3]; and for bovine diseases, the network of animal movements as captured by the Cattle Tracing System [4–6]. More recently, attention has focused on the implications of network structure for human infections transmitted through close contact (such as influenza, SARS and smallpox) using diary-based social encounter information [7], contact tracing [8] or predicted patterns of movements [9] to infer the appropriate network structure. In all cases, it is important to acknowledge the effects of network structure, both because of its impact on the uncontrolled epidemic [10,11] but also because of its utility in targeting controls and tracing the spread of infection [12]. It is, therefore, important that we develop methods of modelling infection dynamics on complex structured networks. Stochastic simulations obviously provide the most accurate and versatile models, but at the expense of tractability. There has, therefore, been considerable focus on developing less computationally intensive approaches that can help us interpret the difference between network-based predictions and those of mean-field (random mixing) models where local structure is ignored.

A recent review paper [13] counterposed three dynamical approaches to the study of epidemic dynamics on networks. These are: pairwise approaches [10], dynamic probability generating function (PGF) formalism [11,14] and heterogeneous mixing [15,16]. Following this approach, we demonstrate here that each of these three approaches is an approximation to the more general pairwise model of Eames & Keeling [17]. We then use the relationships between approaches to derive two new moment-closure-based models for network epidemics: a ‘clustered PGF’ model, which is capable of capturing epidemic dynamics on clustered networks of heterogeneous link distribution using a relatively small number of ordinary differential equations (ODEs), and a ‘heterogeneous susceptible–infectious–susceptible (SIS)' model, which makes a significant but less dramatic reduction of dimensionality where acquired immunity is not long-lasting. Finally, we compare these two models to simulation on exemplar networks similar to those considered in more applied contexts.

## 2. Deriving other approaches from pairwise models

We start by considering a general heterogeneous contact network with *N* nodes. Using the notation developed in Keeling [10] and Eames & Keeling [17], we use square brackets [] to represent the expected numbers of nodes, pairs or triples of any particular type; all notation used is summarized in table 1. The local structure of this network can be defined in terms of three main measures: the degree of distribution *d*_*k*_ (where *d*_*k*_ is the proportion of nodes having *k* network contacts), the clustering of contacts *ϕ* that measures the ratio of triangles (groups of three nodes all connected to each other) in the network to triples of all types (lines of three nodes with or without a transitive link) and the assortativity within the network as captured by the matrix
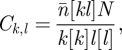
where *n̄* is the mean node degree, which compares the true number of pairs of a given degree with the expected number if half-links connected at random. The matrix 𝒞 represents the extent to which the number of [*kl*] pairs is over- or under-represented with respect to a random process of pairing half-links [18]. We wish to explore how these three forms of local structure influence the types of model formulation that can be successfully applied.

**Table 1.** Notation.

symbol	description
*N*	number of nodes in the network
*M*	maximum node degree
[*k*]	number of nodes of degree *k* (equal to ∑_*A*_ [*A*_*k*_])
[*kl*]	number of pairs with one member having degree *k*, and with the other having degree *l* (equal to ∑_*A*, *B*_[*A*_*k*_*B*_*l*_)
*n̄*	average degree distribution (equal to ∑_*k*_*k*[*k*]/*N*)
*d*_*k*_	proportion of nodes with degree *k* (equal to [*k*]/*N*)
𝒞_*kl*_	correlation matrix between nodes of degree *k* and degree *l*
*ϕ*	clustering coefficient of the network (equal to the number of triangles divided by the number of triples)
[*A*_*k*_]	number of nodes in state *A* with *k* neighbours
[*A*]	number of nodes in state *A* (equal to ∑_*k*_ [*A*_*k*_])
[*A*_*k*_*B*_*l*_]	number of pairs with one member in state *A* and with degree *k*, and with the other member in state *B* and with degree *l*
[*A*_*k*_*B*]	number of pairs with one member in state *A* and with degree *k*, and with the other member in state *B* (equal to ∑_*l*_ [*A*_*k*_*B*_*l*_])
[*AB*]	number of pairs with one member in state *A*, and with the other member in state *B* (equal to ∑_*k*_ [*A*_*k*_*B*])
[*A*_*k*_*B*_*l*_*C*_*m*_]	number of triples with one edge member in state *A* and with degree *k*, with the middle member in state *B* and with degree *l*, and with the other edge member in state *C* and with degree *m*
*θ*(*t*)	the fraction of degree one nodes that remain susceptible at time *t*
*Y*(*t*)	auxiliary variable used in clustered PGF model (equal to ∑_*k*_*k*[*I*_*k*_])
*g*(*x*)	PGF for the network degree distribution (equal to ∑ *d*_*k*_*x*^*k*^)
*τ*	rate of transmission of infection across a network link
*γ*	rate of recovery from infection

The full pairwise equations of Eames & Keeling [17], from which we begin our analysis, are given for SIR-type infections by2.1
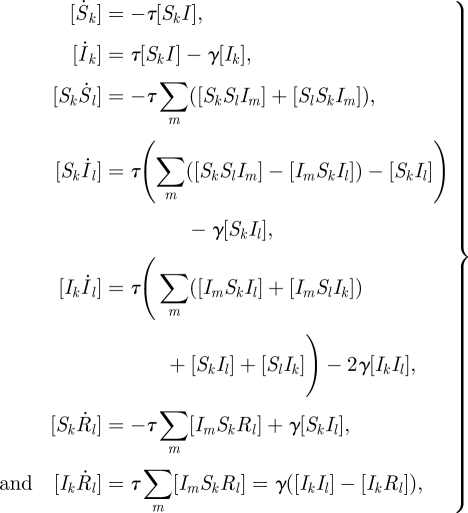
where [*A*_*k*_] refers to the number of nodes of type *A* with degree *k*; a disease state without a subscript implicitly contains the sum over all possible degrees (e.g. [*A*_*k*_*B*] = ∑_*l*_[*A*_*k*_*B*_*l*_]); *τ* is the transmission rate; and *γ* is the recovery rate. (The equations for infections that obey SIS-type dynamics are derived by modifying how recovery acts.) As with many moment-based methods, these equations are exact but unclosed. If we continue to write higher-order equations for triples, quads, etc., then these equations will close only once we reach the network size *N*, destroying the main motivation for the use of ODEs to describe epidemics on networks. We, therefore, must seek approximations that will allow us to close these equations at a lower dimension (approximating the number of triples in terms of pairs and singles), which will aid computation and analytic understanding.

### 2.1. Dynamical and network assumptions

We present now a series of different assumptions about network structure and epidemiological dynamics that allow other approaches to be derived from this general pairwise model, discussing the reasoning behind each assumption and its range of validity. We pay particular attention to the number of equations required to simulate SIR dynamics in terms of the maximum node degree *M*.

#### 2.1.1. Triple closure

Closure schemes for pairwise models, which have become essentially standard, have been presented for networks of significant clustering but with homogeneous degree [10] and also for unclustered networks with heterogeneous degree [17]. The natural combination of these two schemes is2.2
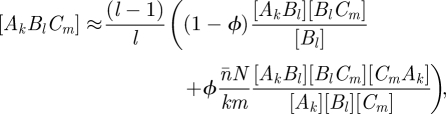
where *A*, *B* and *C* stand for arbitrary disease states. While this closure is the natural extension of existing approximations, it does pose a question of interpretation compared to existing closures, since the term proportional to *ϕ* cannot be rigorously interpreted as a prevalence of triangles owing to the lack of symmetry between the *k*, *l* and *m* nodes. An extended discussion of how to interpret asymmetric clustered closure appears in House & Keeling [19]. It is also worth noting that the unclustered pairwise equations can be reinterpreted in terms of neighbourhoods [20], although this approach has yet to be extended to the clustered case.

The closure scheme (2.2) acting on the full pairwise equations (2.1) produces a closed ODE system of (5*M* + 2)*M* independent equations for SIR dynamics, which can quickly outstrip even modern computational resources for graphs with ‘fat tailed’ degree distributions such as scale-free networks, where *M* is often very large. This motivates the investigation of further approximations that can allow us to reduce the system size.

One additional, commonly made assumption that will be used in deriving other approaches is the absence of triangles in the network, that is,2.3
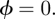


#### 2.1.2. Deconvolution of pairs

The assumption introduced by Eames & Keeling [17] was to express the joint probability of a fully described pair as a product involving pair types and network structure:2.4
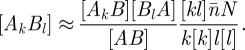


Under this assumption, pairs with a single indexing degree are the main variables, which significantly reduces the number of independent ODEs needed in the pairwise equations to 10*M* for SIR dynamics. *A* *priori* the accuracy of this assumption is not assured, since it depends on the precise dynamical process taking place on the network; however, for all epidemiological scenarios so far considered in the literature, the results using this approximation are in extremely close quantitative agreement with the full pairwise model. Most importantly, this assumption allows the consideration of assortative mixing and heterogeneous degree, in a system where a number of differential equations is linear rather than quadratic in the maximum node degree.

#### 2.1.3. Detailed balance

The most general prevalence of pairs of connected nodes with degrees *k* and *l* is given by
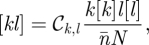
that is, if 𝒞_*k*, *l*_ can take any value for combinations of *k* and *l*, then all possible distributions for [*kl*] can be enumerated. However, an assumption is often made that the correlation matrix 𝒞 obeys2.5
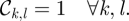


In mean-field (non-network) models, such an assumption has been used, to great effect, to study the spread of sexually transmitted infections in risk-structured populations defined by individual-level data on sexual contacts [21]; however, it is recognized that, in general, most populations are assortative such that 𝒞_*k*, *l*_ > 1 for similar *k*, *l* and 𝒞_*k*,*l*_ < 1 for dissimilar *k*, *l*. In network models, the assumption that 𝒞_*k*, *l*_ = 1 means that link ‘stubs’ from each node are connected randomly, which is typically called *detailed balance* with respect to swapping randomly picked edges in physical science [18], although terminology and definitions concerning assortativity can be different in other subject areas, for example, the sexually transmitted infection literature (e.g. [22]).

#### 2.1.4. Pair closure

If network dynamics are effectively dominated by assortative mixing of risk classes, then we may wish to remove pair-level variables through the assumption2.6
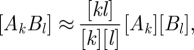
which allows us to keep assortativity but loses the effects of network structure—the correlation between the states of connected nodes is lost—although some of these can be maintained through extra factors, as in Kiss *et al.* [23]. Application of this closure reduces the number of equations needed to 2 *M* for SIR dynamics.

#### 2.1.5. Deconvolution of individuals

The final approximation we consider has not been previously explicitly stated in this form, but can be used to derive the PGF approach from a general pairwise model. It can be viewed as the main assumption that allows the PGF formalism to approximate disease dynamics on heterogeneous networks within a low dimensional framework. This involves writing the joint probability [*A*_*k*_*B*] (defined above) as a product, and is analogous to the concepts used to generate equation (2.4), in that its validity depends on the independence of dynamics and network structure.2.7
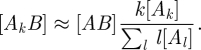


The underlying approximation is that given a node (of type *A*_*k*_), the type of connected node (taken here to be *B*) is independent of *k* and therefore independent of the local network structure. For SIR dynamics, this assumption creates a set of equations whose dimension does not depend on the maximum node degree *M*, although the exact number of equations required depends on whether clustering is present, and which quantities one wishes to calculate over the course of an epidemic.

### 2.2. Relating ordinary differential equation-based approaches

Starting with the general pairwise model (2.1), the assumptions above can be used to derive other approaches. Taking the assumptions (2.2)–(2.5) and (2.7) allows us to derive the PGF equations originally formulated from first principles in [14] (full equations given in electronic supplementary material). This is an interesting and unexpected result since the underlying arguments used originally to derive the pairwise and PGF models are quite different. The standard heterogeneous-mixing models associated with risk-structured populations (given in electronic supplementary material) are derived by putting the assumption (2.6) into the pairwise equations and ignoring the pair-level variables.

We also note that for networks with no clustering and degenerate degree distribution (often called either regular graphs or homogeneous random networks), the PGF and pairwise approaches are formally identical, since assumption (2.7) is trivially satisfied.

## 3. Construction of novel models

While the formal links between prima facie distinct epidemic models are intrinsically interesting, the main motivation for our work is to derive novel, parsimonious models for epidemics on a range of complex networks without having to argue the formulation from first principles. The fact that full pairwise models are simply written down, together with the new explicit form of the assumption underlying PGF models (2.7), means that it is possible to produce systematically much simpler ODE-based models for network-based epidemics. We now consider two such models, together with the potential limits on this methodology.

### 3.1. Incorporation of clustering

The relationships between different ODE approaches to network epidemics presented above open up the intriguing possibility of extending the PGF approach to include clustering so that both heterogeneity in link distribution and clustering can be analysed using a low-dimensional model with a small number of dynamical variables. This is indeed possible: using (2.4) and (2.7) together with the standard pairwise closure (2.2), the two triples that appear in the unclosed pairwise SIR equations can be closed through3.1
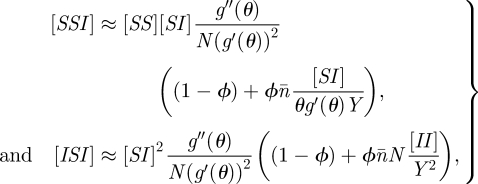
where we define *θ*(*t*) as the fraction of degree 1 nodes that remain susceptible at time *t* and3.2
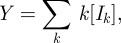
and *g*(*x*) = ∑_*k*_ *d*_*k*_ *x*^*k*^ is the PGF for the node degree distribution. This gives a new dynamical system that, together with the closure relations (3.1), determines the epidemic behaviour:3.3
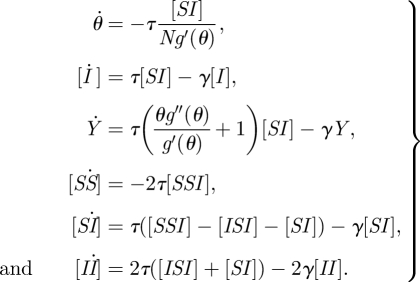


The number of susceptibles can be tracked non-dynamically through the relationship3.4
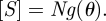


The primary significance of this result is that, regardless of the maximum node degree on a network, a system of six ODEs together with the non-dynamical relationship between [*S*] and *θ* can be used to calculate an expected prevalence curve for heterogeneous, clustered networks.

### 3.2. Incorporation of other disease natural histories

In addition to the clustered PGF model derived above, which assumes SIR (susceptible–infectious–recovered) dynamics, other extensions of the PGF approach will clearly be possible, for example, the inclusion of a latent class or multiple infectious classes as in Kamp [24]. While this requires more variables, the system will still be of far lower dimension than the equivalent full pairwise model.

However, other commonly studied disease behaviours, such as the SIS paradigm, which is ideal for sexually transmitted infections and has been extensively studied using pairwise approaches, are not straightforwardly reduced to the PGF formulation. This is because, under SIS dynamics, the set of ODEs governing the evolution of quantities such as ∑_*k*_*k*^*a*^ [*A*_*k*_] is not closed. This does not, however, preclude the use of approximation (2.7). Inserting this assumption into the standard pairwise SIS model gives the following new model:3.5
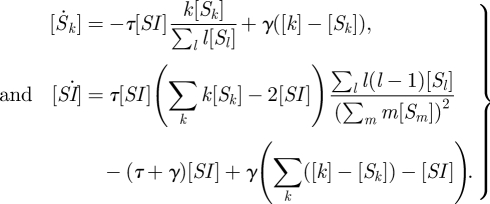


Unlike the clustered PGF model above, the number of these equations that need to be manipulated numerically is *M* + 1, that is, linear in the maximum node degree; however, their dimensionality is still significantly lower than standard pairwise models and, perhaps more importantly, there are only two equations that need to be manipulated in analytic work on this system.

### 3.3. Assortativity

Deviation from null assortativity is a complexity that simply cannot be naturally incorporated in the PGF equations without vastly increasing the dimensionality of the system. We provide a numerical demonstration of the importance of this observation in the electronic supplementary material. Nevertheless, as shown in Eames & Keeling [17], by using networks (2.2) and (2.4), an assortative model can be created that is linear in maximum node degree.

### 3.4. Comparison with simulation

We now test each of the two new models above (clustered PGF and heterogeneous SIS) against simulation and compare to other relevant ODE approaches.

**Figure 1.**
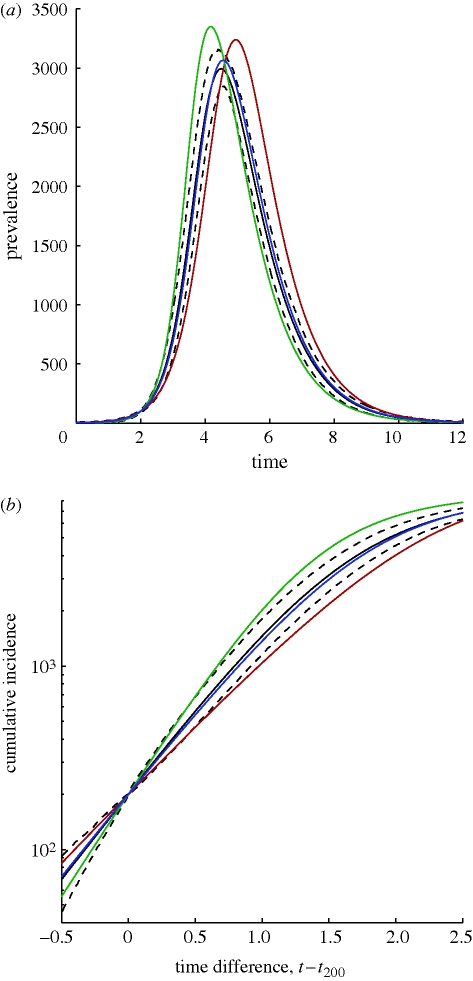
Numerical test of the clustered PGF model against simulation and other ODE approaches. The network has size *N* ≈ 10^4^, its degree of distribution is Poisson with mean *n̄* = 6 and the clustering coefficient is *ϕ* = 0.2. The transmission rate is *τ* = 0.8 at unit recovery rate. We shift time for each of 10^3^ stochastic simulations, so all curves agree on when a cumulative incidence of 200 is reached, and the simulation mean and prediction interval can be meaningfully visualized. Clearly, the clustered PGF approach is in excellent agreement with simulation. Solid line, simulation mean; dashed line, simulation 95% PI; red line, homogeneous pairwise; green line, PGF; blue line, clustered PGF.

For the clustered PGF, we start with a random graph of approximately 10^4^ nodes and with Poisson parameter *λ* = 6. We then introduce a clustering coefficient of *ϕ* = 0.2 using the ‘big V’ rewiring [19,25], which does not change the degree distribution. Following [26], we bias node selection by *k*(*k* − 1), so individual-level clustering is constant; while this will make *C*_*kl*_ slightly deviate from unity, at the level of clustering we consider this should have negligible dynamical impact. We choose these network parameters because they separate the ODE approaches without being so large that concerns about global network properties like giant component size are posed. While it is probable that realistic networks for respiratory infection have more variable degree distributions, larger numbers of mean nodes and higher clustering coefficients, our approach is to start with a system with few underlying parameters and a transparent method for the introduction of clustering. Epidemic rates were *τ* = 0.8, *γ* = 1. Figure 1 shows the comparison of the clustered PGF model, which is in good agreement with simulation, and two other ODE approaches. The homogeneous pairwise model underestimates early growth in cases and, conversely, the unclustered PGF model overestimates early growth. This is as would be expected from general arguments about the effects of clustering [10] and population heterogeneity [15]. The electronic supplementary material shows how these other, existing, ODE approaches remain in good agreement with simulations on appropriate networks and also considers the impact of assortativity.

**Figure 2.**
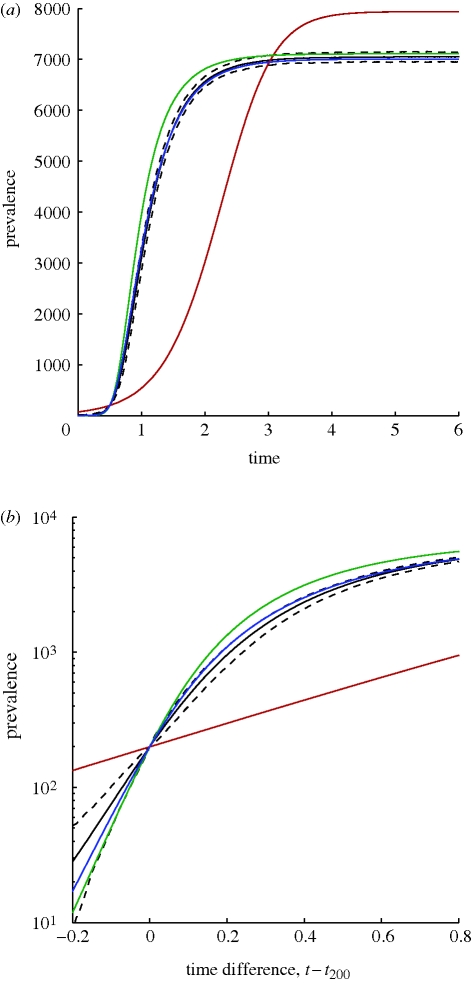
Numerical test of the heterogeneous SIS model and other ODE approaches. The network has size *N* ≈ 10^4^, and is scale-free with parameters *m*_0_ = 20 and *m* = 2. The transmission rate is *τ* = 1.0 at unit recovery rate. We shift time for each of 10^3^ stochastic simulations, so all curves agree on when a prevalence of 200 is reached, and the simulation mean and prediction interval can be meaningfully visualized. Clearly, the heterogeneous pairwise approach is in excellent agreement with simulation. Solid line, simulation mean; dashed line, simulation 95% PI; red line, homogeneous pairwise; green line, heterogeneous mixing; blue line, heterogeneous pairwise.

For the heterogeneous SIS model, we generate a scale-free network of approximately 10^4^ nodes by using the standard [27] method with parameters *m*_0_ = 20, *m* = 2, and removing nodes of degree 0. Since the scale-free property is consistent with observed sexual contact networks [28], and for sexually transmitted infections recovered individuals often fail to acquire long-lasting immunity, pairing scale-free networks with SIS dynamics is natural. Epidemic rates were *τ* = 1, *γ* = 1. Figure 2 shows that models incorporating heterogeneity both fit to simulation much better than the homogeneous pairwise model and that the addition of just one extra equation for the heterogeneous pairwise when compared with heterogeneous mixing significantly improves the fit to early growth behaviour.

## 4. Discussion

We have analysed the conditions under which PGF and heterogeneous-mixing models can be derived from a general pairwise approach. These split into assumptions about the network itself, such as zero clustering (2.3) and non-assortativity (2.5), and assumptions about the interaction of dynamics with network structures such as (2.2), (2.4), (2.6) and (2.7). We have used these conditions to derive a clustered PGF model and a low-dimensional heterogeneous SIS model, which are likely to be of significant utility in the study of epidemics on networks owing to their relatively low dimensionality.

In general, the starting point for analysis of disease transmission on a network has to be the available data. Where the full network is known, or can be imputed with confidence, then explicit stochastic simulation of the epidemic process is likely to be the best approach. When only statistical properties of the network, such as degree distribution *d*_*k*_, correlation between degrees 𝒞_*kl*_ or clustering coefficient *ϕ*, are known, then there are two complementary approaches: either generate exemplar networks for simulation with the appropriate statistics or make use of ODE-based models of the kind considered here, which can be parametrized directly from the network statistics, are numerically tractable and mathematically transparent.

Within this second approach, each ODE-based model used to study network epidemics will have its own domain of validity. While the most general pairwise model can be applied to all compartmental paradigms, with clustering, degree heterogeneity and assortativity included, this comes with increased and potentially unnecessary computational overhead. The more tractable models, on the other hand, may suffer from important inaccuracies when the implicit assumptions they make are not justified.

A question is also posed, however, about the ultimate justification of even the general pairwise model. At present, it is widely believed that in an appropriate regime, this model will be ‘exact’ in the same way that the standard SIR equations for an epidemic tend to the mean behaviour of an exact stochastic epidemic model in the appropriately constructed large-population limit. Whether such a regime can be defined remains an important open problem, and one that will hopefully be resolved in the near future.

In conclusion, we hope that this study has clarified the relationship between diverse ODE-based models and extended the repertoire of models available for use.
